# Mental Health Crisis and Stress Coping among Healthcare College Students Momentarily Displaced from Their Campus Community Because of COVID-19 Restrictions in Japan

**DOI:** 10.3390/ijerph18147245

**Published:** 2021-07-06

**Authors:** Masatoshi Tahara, Yuki Mashizume, Kayoko Takahashi

**Affiliations:** 1 Graduate School of Medical Sciences, Kitasato University, 1-15-1, Kitasato, Minami-ku, Sagamihara 252-0373, Kanagawa, Japan; dm18026@st.kitasato-u.ac.jp (Y.M.); kayo.ot@kitasato-u.ac.jp (K.T.); 2 Department of Rehabilitation Therapist, Saiseikai Higashikanagawa Rehabilitation Hospital, 1-13-10, Nishikanagawa, Kanagawa-ku, Yokohama 221-0822, Kanagawa, Japan; 3 Department of Occupational Therapy, School of Allied Health Sciences, Kitasato University, 1-15-1, Kitasato, Minami-ku, Sagamihara 252-0373, Kanagawa, Japan

**Keywords:** COVID-19, mental health, stress coping, healthcare college students

## Abstract

College students are one of the most affected groups by self-quarantine due to COVID-19, as they may live in loneliness and anxiety, increasing their risk of mental health crisis. This study aimed to identify risk factors for poor mental health and stress coping strategies among healthcare college students during the COVID-19 pandemic in Japan. A cross-sectional survey was conducted over 7 consecutive days starting on 28 April 2020 using a web-based questionnaire. The survey assessed socioeconomic characteristics and the General Health Questionnaire-12 score, self-reported health status, anxiety, and satisfaction with daily life, work, leisure, and new activities. Approximately 70% of 223 respondents had poor mental health. Less communication with friends was the main risk factor for mental health problems. Good health status and satisfaction with leisure and new activities were associated with reduced risk of mental health problems. Students with poor mental health tended to seek social support as a stress coping strategy. This study showed that the mental health of students declined during self-quarantine, and loneliness could be the major reason. There is a need for a new form of communication and learning that deals with the isolation and loneliness of students, especially for students living alone.

## 1. Introduction

The impact of the 2019 coronavirus disease (COVID-19), which began in China and spread around the world starting in December 2019 [1], has profoundly altered the economic activities and daily lives of people everywhere [2]. Increased stress and loneliness due to COVID-19 and the measures taken to slow the spread of COVID-19 have led to the deterioration of mental health in the general public and particularly in certain vulnerable subpopulations, such as students [3].

COVID-19 has resulted in lost learning opportunities and economic stress for college students [4,5]. About 30% of college students were reported to have mental health problems such as depression and anxiety due to the spread of COVID-19 [6,7]. In Japan, the government asked primary and secondary schools across the nation to close temporarily on 2 March 2020. Nine days later, the World Health Organization declared COVID-19 a pandemic [8]. On 7 April 2020, the Japanese government issued an emergency declaration and designated 13 prefectures, including Tokyo and Osaka, as special caution areas [9]. That declaration required people to reduce interpersonal contact by 80% and to limit outings; it changed the lifestyles of many people [10], which meant that many students could no longer go to school. In addition, especially for students living alone, going to school and going out, such as shopping, were restricted, which meant spending time alone at home.

For college students, participation in the college community provides a foundation for spiritual growth. Online lessons alone cannot compensate for the loss of interactions with that community. Students whose engagement with the college community is limited to online lessons at home alone are vulnerable to feelings of loneliness and anxiety, which adversely affect mental health. Therefore, it is not enough to deal with the loss of learning opportunities; it is also necessary to understand the mental vulnerabilities caused by the loss of community. In addition, it is necessary to consider specific risk factors and coping strategies that affect the deterioration of mental health. In particular, healthcare college students are at risk of worsening mental health compared to other college students, as are healthcare workers exposed to infection and social risks during a pandemic and a special work environment different from the general public [11,12,13].

Coping strategies, defined as a person’s constantly changing cognitive and behavioral efforts to manage specific external and/or internal demands, are indispensable in stressful circumstances [14,15]. Previous studies reported on the effects of COVID-19 restrictions on students’ mental health [2] but did not provide any guidance on coping strategies to deal with them. Therefore, it is useful to investigate how students spend their lives and cope with stress in a lifestyle that has been transformed by COVID-19 to support their mental health in the COVID-19 pandemic.

We conducted a cross-sectional observational investigation of healthcare college students to identify specific risk factors and coping strategies that affect the mental health of COVID-19-affected college students.

## 2. Materials and Methods

We conducted a cross-sectional survey of third-year and fourth-year students at Kitasato University School of Allied Health Sciences using a web-based questionnaire (Google Form) from 28 April 2020 to 4 May 2020. Considering the mental burden on participants living in a pandemic to answer questions, they were not prompted for answers and were not linked to individuals to prevent duplicate answers.

Survey items included demographic information such as age, gender, number of people living together, region of residence, year of university, communication with friends and family, and financial status. Regions of residence were divided into the alert region (13 prefectures of the special alert area) and the non-alert region (the remaining 32 prefectures in Japan). Communication with family and friends was rated on a three-point Likert scale: “same as usual”, “less than usual”, and “more than usual”. The respondents’ financial situation was also rated on a three-point Likert scale: “no change”, “worse than usual”, and “better than usual”.

We assessed mental health status using the Japanese version of The General Health Questionnaire-12 (GHQ-12), which includes 12 questions regarding mental health status, with four options for each question. The GHQ-12 typically has two scoring methods, bimodal (0-0-1-1) and Likert scale (0-1-2-3) [16]. We used bimodal scoring in the present study. In accordance with previous studies, we considered a GHQ-12 score ≥ 4 as indicative of mental health problems [17,18].

We investigated health status and anxiety over COVID-19 using a visual analog scale, with scores ranging from 1 (“not at all healthy” or “not at all anxious”) to 10 (“very healthy” or “very anxious”). To capture satisfaction with daily life, we developed an original 10-point Likert scale based on the Canadian Occupational Performance Measure [19], which included satisfaction with leisure, satisfaction with job, satisfaction with daily-life activities, and satisfaction with new activities started since COVID-19 restrictions began [20,21]. A score of 1 indicated “not satisfied at all” or “no new activities”. A score of 10 indicated “very satisfied”. Participants were also asked to give an example of a new activity started since COVID-19 restrictions began.

Different types of behavioral motivation are related to stress and wellbeing [22]. We asked participants about their motivation for self-restraint regarding COVID-19. The participants indicated on a visual analog scale the degree to which their motivation was due to intrinsic factors, such as not wanting to spread infection, or to extrinsic factors, such as requests from the government. A score of 1 indicated “self-determination based on extrinsic motivation”. A score of 10 indicated “self-determination based on intrinsic motivation”.

Since the spread of COVID-19 in Japan and the issuance of a state of emergency were completely unexpected to us, and the period was not clarified, these survey items have not been surveyed in advance.

To evaluate the respondents’ coping strategies, we categorized behavioral efforts to manage stress into classes used in previous research [23], which included confrontive coping, distancing, self-control, seeking social support, accepting responsibility, escape/avoidance, planful problem solving, and positive reappraisal. Confrontive coping includes aggressive efforts to alter the situation and involves a degree of hostility and risk taking. Distancing includes efforts to detach oneself from the adverse situation. Self-control includes efforts to regulate one’s own feelings and actions. Seeking social support includes efforts to seek informational support, tangible support, and emotional support. Accepting responsibility means acknowledging one’s own role in the problem with a concomitant theme of trying to put things right. Escape-avoidance includes wishful thinking and behavioral efforts to escape or avoid the adverse situation. Planful problem solving includes deliberate problem-focused efforts to alter the situation coupled with an analytic approach to solving the problem. Positive reappraisal includes efforts to create positive meaning by focusing on personal growth. We determined which coping strategies each respondent used and calculated the overall use of each category among all the students.

We divided the respondents according to mental health status into a poor mental health group (GHQ-12 ≧ 4) and a normal group (GHQ-12 < 4). We performed Mann–Whitney tests using SPSS software (version 25, IBM Corp.) to compare subgroups based on mental health status, gender, year of university, and number of household members. We calculated the adjusted odds ratio (OR) with a 95% confidence interval (CI) for risk of poor mental (GHQ-12 ≧ 4) health associated with COVID-19 restriction. The multiple logistic regression analysis performed with adjustments for all potential factors as listed in Table 1 and Table 2. We used *p* < 0.05 as the threshold for significance in all statistical tests.

## 3. Results

Of 828 eligible students, a total of 226 (27.3%) healthcare college students responded to the survey for 7 days from 24 April 2020 to 4 May 2020. Three respondents did not complete the survey and were excluded from the analysis.

### 3.1. Demographic Characteristics

The demographic data of the 223 (26.9%) respondents that completed the survey are shown in Table 1. The participants included 175 females (78.5%). The age distribution was as follows: 20 years, 92 participants (41.3%); 21 years, 103 participants (46.2%); 22 years, 24 participants (10.8%); 23 years, 3 participants (1.3%); and 24 years, 1 participant (0.4%). A total of 121 participants (54.3%) were third grade students, and 102 participants (45.7%) were fourth grade students. In terms of living status, 81 (36.3%) lived alone, and 142 (63.7%) lived with two or more other people. A total of 197 participants (88.3%) lived in the alert region and 26 (11.7%) lived in the non-alert region.

Regarding financial situation, 112 participants (50.2%) answered “no change”, and 91 participants (40.8%) answered “worse than usual” and 20 participants (9.0%) answered “better than usual”. For amount of communication with family, 86 participants (38.6%) answered “same as usual”, and 11 participants (4.9%) answered “less than usual”, and 126 participants (56.5%) answered “more than usual”. Conversely, 22 participants (9.9%) answered “same as usual”, and 192 (86.1%) responded “less than usual”, and 9 participants (4.0%) answered “more than usual” for amount of communication with friends.

### 3.2. Severity of Mental Health Problems and Associated Factors

Table 2 shows the medians, interquartile ranges (IQRs), and statistical comparisons of the GHQ-12, anxiety, health, self-determination, and satisfaction scores stratified by gender, year of university, and number of household residents. The Median score of GHQ-12 was 5 (interquartile range (IQR): 3–8). The other Median scores were 8 (IQR: 7–10) for anxiety over COVID-19, 8 (IQR: 6–10) for health condition, 7 (IQR: 5–9) for self-determination score, 3 (IQR: 1–4) for satisfaction with leisure, 4 (IQR: 2–6) for satisfaction with study, 4 (IQR: 3–7) for satisfaction with daily life activities, and 6 (IQR: 2–8) for satisfaction with new activities started since social distancing began.

Compared with men, women had higher anxiety regarding COVID-19 and lower satisfaction with daily-life activities (*p* = 0.001 and *p* = 0.04, respectively). Students in their third year of university were slightly more satisfied with their leisure activities than students in their fourth year (*p* = 0.02), although satisfaction with leisure activities was low in both groups. Compared with students who lived with other individuals, students who lived alone reported lower satisfaction with their studies and new activities (*p* = 0.03 for both comparisons).

Table 3 shows demographic characteristics and mental health measurements for normal and severe mental health problems groups. The severe mental health problems group (GHQ-12 score ≥ 4) including 158 participants (70.9%) exhibited these characteristics: 128 (81.0%) were females; 83 participants (52.5%) were third grade students, and 75 participants (47.5%) participants were fourth grade students; 60 (38.0%) lived alone, and 98 (62.0%) lived with two or more other people; 144 participants (91.1%) lived in an alert region, and 14 participants (8.9%) lived in a non-alert region; 73 (46.2%) answered “same as usual”, and 71 (44.9%) responded “worse than usual” for financial situation; 58 (36.7%) answered “no change”, and 9 (5.7%) responded “less than usual” for communication with family; and 7 (4.4%) answered “same as usual”, and 144 (91.1%) responded “less than usual” for communication with friends.

Compared with the students with normal mental health status, the students with poor mental health status had less anxiety about COVD-19 (*p* = 0.03), higher self-reported health status (*p* < 0.001), higher self-determination scores (*p* = 0.04), and greater satisfaction with leisure (*p* < 0.001), studies (*p* = 0.001), daily-life activities (*p* < 0.001), and new activities (*p* < 0.001; Table 4).

### 3.3. Resilience Factors for Mental Health Outcomes

After adjustment for covariates in the logistic regression analysis, less communication with friends (odds ratio (OR), 5.38; 95% confidence interval (CI), 1.54–18.83; *p* = 0.008) was independently associated with increased risk of mental health problems (Table 5). By contrast, good health status (OR, 0.68; 95% CI, 0.55–0.83; *p* < 0.001), satisfaction with leisure (OR, 0.68; 95% CI, 0.58–0.79; *p* < 0.001), and satisfaction with new activities (OR, 0.88; 95% CI, 0.78–0.99; *p* = 0.04) were associated with reduced risk of mental health problems.

### 3.4. Coping Strategies: New Activities Initiated since Social Distancing Began

**Figure 1:**
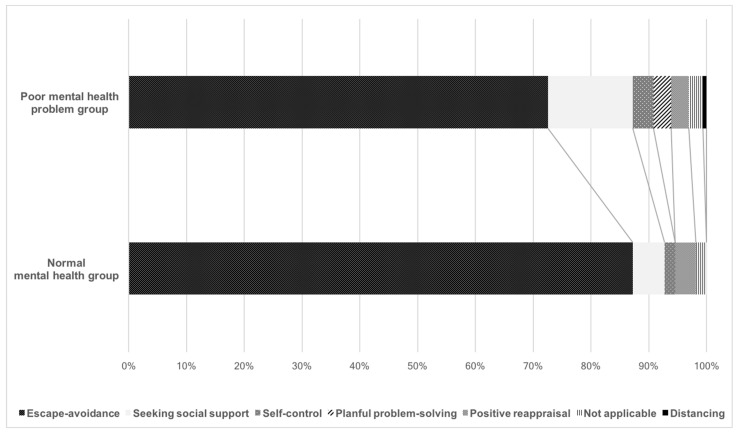
Coping strategies classified using descriptive data according to differences in mental health status.

Figure 1 shows the types of coping strategies reported by the students. Thirty-five of 65 respondents (53.8%) with normal mental health and 98 of 158 respondents with poor mental health (62.0%) reported some kind of stress coping. Figure 1 contrasts the poor mental health problem group with the normal mental health group, and shows the contrast of the adoption rate of each coping style with a line. Escape/avoidance was the most common coping strategy reported by students in both groups. The next most common strategy in both groups was seeking social support, which was more common among students with poor mental health than among students with normal mental health.

## 4. Discussion

### 4.1. Factors Contributing to Mental Health Decline among Healthcare College Students

The influence of COVID-19 on the mental health of healthcare college students is serious, and support for mental health is necessary [24]. The GHQ-12 scores indicated that 70.9% of the respondents had poor mental health, which is higher than before the pandemic [25,26]. Female students were more anxious about COVID-19 than male students. Previous research suggested that female students were more likely to have mental health problems than male students before the pandemic [27]. It possible that female students experienced more anxiety due to COVID-19 than male students, resulting in more extensive self-quarantine and lower satisfaction with daily-life activities. In addition, previous studies [28] have reported that different races and incomes have different effects on mental health during a pandemic, but this study was conducted at a private university with Japanese subjects. Therefore, the impact of race and income was not considered in the discussion. Fourth-year students had lower satisfaction with leisure than third-year students. The reason for that might be that the fourth-year students go to practical training in hospitals and nursing homes and might therefore have had stronger self-quarantine motives to try to prevent the spread of infection. In addition, the fourth-year students may have been influenced by the professional ethics peculiar to medical students who aspire to be medical staff. Because they are more trained in the clinical setting than students in other vocations, they know their professional attitude and the rigor of infection control.

Students who lived alone had lower satisfaction with studies and new activities than students who lived with others. Because of the spread of COVID-19, the students were switched to online classes. Students living alone could not fully prepare for the learning environment, such as the stable internet and personal computers. This is because many students living alone used school computers and the Internet environment to search documents and write reports. In addition, students living alone often live in relatively small apartments, further limiting their opportunities for in-home exercise and new activities. Moreover, students living with two or more can start new activities jointly, while students living alone have difficulty choosing such interactive activities.

Compared with the students with normal mental health, those with poor mental health had lower life satisfaction, lower health status, higher COVID-19 anxiety, and a higher degree of external motivation. These comparisons suggested that the deterioration of participants’ mental health may be the result of being “reactive”, that is, a stress response to restricted activity and anxiety over infection [29]. In addition, previous studies suggested that internal motivation for self-determination is associated with wellbeing [22,30,31]. It is possible that the tendency for self-determination based on external, rather than internal, motivation led to a decrease in life satisfaction and deterioration of mental health.

In addition, it is necessary to mention the peculiarity that the participants of this study are university students of medical universities. During the period of this study, when the Government of Japan issued the first state of emergency, all citizens were requested to refrain from going out. Therefore, participants in this study were restricted from attending school, and there was little difference in the risk of exposure to COVID-19 compared to students other than medical college students. However, medical college students may work in the medical field in the near future. Therefore, participants may be more likely to be afraid of infection than other college students, just as medical staff are afraid of infection and related proceedings [32].

### 4.2. Risk and Resilience Factors

Lack of communication with friends was a risk factor for poor mental health. In previous studies, loneliness was associated with mental health problems such as depression [33]. Conversely, students with good health status and high satisfaction with leisure and new activities were more resilient in terms of mental health than students with poor health status and low satisfaction with leisure and new activities. Previous studies reported a relationship between physical health status and mental health status [34,35]. In a pandemic situation, it is difficult to maintain an existing lifestyle. Our results showed that satisfaction with daily life declined since the beginning of COVID-19 restrictions. Therefore, it might be possible to alleviate mental health problems by engaging in new activities, improving leisure satisfaction, and adapting to new lifestyles.

### 4.3. Stress Coping Strategies

Most of the respondents used escape/avoidance to deal with the pandemic. Previous research showed that escape/avoidance is adopted in uncontrolled situations [14]. In addition, escape/avoidance stress coping tended to be used in the COVID-19 pandemic [19]. The self-quarantine associated with COVID-19 is a situation that cannot be controlled by individuals, which explains why most of the respondents adopted the escape/avoidance coping strategy.

Some of the students with poor mental health reported seeking social support. Women, especially, tend to choose external coping strategies such as seeking social support [35]. In addition, the seeking social support stress coping tended to be adapted by poor mental health people in the COVID-19 situation [19]. It is possible to maintain mental health by recognizing social support in the event of a disaster [36]. COVID-19 is a unique situation, however, and it is difficult for students to obtain accurate and appropriate information about social support and infection control. The dissemination of inaccurate information might have increased anxiety and contributed to poor mental health. Furthermore, during self-quarantine, the means of gathering information were limited to the internet and television. A previous study reported deterioration of mental health due to long-term use of the internet [37,38].

The mental health deterioration among college students during COVID-19 was largely related to momentary displacement from the campus community. Of course, as a stressor in the COVID-19 pandemic, there is a good possibility that various factors such as economic conditions and health conditions [39] were intricately intertwined. In addition, the results of this study extracted factors such as loneliness related to temporary inability to attend school. Women have more psychological development than men in terms of mature interpersonal relationships [40], so the loss of community, which is important for developing interpersonal relationships, might have had a greater impact on women than on men. Momentary displacement from the campus community leads to deterioration of mental health, especially for individuals living alone. Loneliness and anxiety might make it harder to obtain accurate information about COVID-19 and other important topics, which might explain why students living alone tended to seek social support.

Eliminating self-quarantine and allowing students to come to college might be one way to prevent further mental health deterioration during the pandemic. That approach is impractical, however, because of the risk of spreading COVID-19. Although existing resources are scarce, especially in a serious pandemic such as COVID-19, there are various support methods to alleviate mental problems such as remote support using online [41]. Therefore, it might be possible to set up time for online group discussions to promote student interaction. Allowing partial school attendance might be a way to facilitate participation in the student community while reducing the risk of domestic infection, especially for students living alone. Flexible measures that take into account the university community and the role of the community are necessary to support university students who are at risk of mental health deterioration.

### 4.4. Limitations

Our results might be affected by selection bias and also regional bias. The target university is in the alert region, and 88.3% of participants lived in the alert region, which does not reflect the population of rural areas. In addition, the study was limited to 7 days and could not track long-term mental health problems. Additional longitudinal studies are needed to investigate changes in mental health status in healthcare college students over time. One of the limitations of our research is the low response rate and the small number of samples. The reason for the low response rate and low number of participants was that there was only one university recruited, and there were few reminders. In addition, since no measures have been taken to prevent duplicate answers, the possibility of these problems cannot be ruled out, which is also a limitation of this study. In addition, because it was a cross-sectional survey, participants did not know their previous mental health status and did not know the baseline of their responsiveness to changes in their lives.

## 5. Conclusions

Over half of surveyed healthcare college students reported mental health problems since the start of COVID-19 restrictions in Japan. Less communication with friends was a risk factor for poor mental health. Participation in leisure and new activities is a proactive strategy that supports good mental health during the pandemic. Students with poor mental health status were more likely than those with normal mental health status to adopt social support seeking coping for stress coping. The results of this study suggested that there is a need for opportunities for mutual communication between students and the new form of learning that deals with the isolation and loneliness of students, especially considering students living alone during the COVID-19 restrictions.

## Figures and Tables

**Table 1:** Demographic characteristic of the study participations (n = 223).

Characteristic	Number	Percentage
Gender		
Male	48	21.5
Female	175	78.5
Age, years		
20	92	41.3
21	103	46.2
22	24	10.8
23	3	1.3
24	1	0.4
Year of university		
Third	121	54.3
Fourth	102	45.7
Place of residence		
alert region *	197	88.3
Non-alert region	26	11.7
Household members		
One	81	36.3
Two or more	142	63.7
Economic situation		
Same as usual	112	50.2
Worse than usual	91	40.8
Better than usual	20	9.0
Communication with family		
Same as usual	86	38.6
Less than usual	11	4.9
More than usual	126	56.5
Communication with friends		
Same as usual	22	9.9
Less than usual	192	86.1
More than usual	9	4.0

* A total of 13 prefectures were designated as alert regions.

**Table 2:** Survey responses of the study participants.

	Total	Gender			Year of University			Household Members		
Measurements		Male	Female	*p* Value	Third	Fourth	*p* Value	One	Two or More	*p* Value
GHQ-12	5 (3-8)	5 (2–7)	6 (3–8)	0.26	5 (2–8)	6 (3–8)	0.22	6 (3–8)	5 (3–8)	0.25
Anxiety over COVID-19	8 (7–10)	7 (5.75–9)	8 (7–10)	0.001	8 (7–10)	8 (7–9)	0.19	8 (7–9)	8 (7–10)	0.35
Health condition	8 (6–10)	8 (7–10)	8 (6–9)	0.15	8 (6–9)	8 (7–10)	0.49	7 (5–8)	7 (5–9)	0.80
Self-determination score	7 (5–9)	7 (4.75–9)	7 (5–9)	0.33	8 (5–9)	7 (5–9)	0.44	7 (5–9)	7 (5–9)	0.67
Satisfaction with daily life										
Leisure	3 (1–4)	2.5 (1–5)	3 (1–4)	0.72	3 (2–5)	2 (1–4)	0.02	3 (1–4)	3 (2–5)	0.21
Study	4 (2–6)	4 (2–7)	4 (2–6)	0.46	4 (2–7)	3.5 (2–5)	0.15	3 (2–6)	4 (2.25–6)	0.03
Daily-life activities	4 (3–7)	6 (3–7)	4 (3–6)	0.04	4 (3–7)	4 (3–6.75)	0.92	4 (3–6)	4 (3–7)	0.46
New activities	6 (2–8)	6 (3–8)	6 (2–8)	0.24	7 (3–8)	6 (1–8)	0.08	6(1–8)	7 (3.25–8)	0.03

All values given as the median (IQR). *p* values correspond to Mann–Whitney U test. Abbreviation: IQR = interquartile range, GHQ-12 = General Health Questionnaire 12.

**Table 3:** Demographic characteristics of students with normal and poor mental health status.

	Normal (n = 65)	Poor (n = 158)
Gender		
Male	18 (27.7)	30 (19.0)
Female	47 (72.3)	128 (81.0)
Age, years		
20	30 (46.2)	62 (39.2)
21	27 (41.5)	76 (48.1)
22	6 (9.2)	18 (11.4)
23	2 (3.1)	1 (0.6)
24	0 (0.0)	1 (0.6)
Year of university		
Third	38 (58.5)	83 (52.5)
Fourth	27 (41.5)	75 (47.5)
Place of residence		
Alert region *	54 (83.1)	144 (91.1)
Non-alert region	11 (16.9)	14 (8.9)
Household members		
One	21 (32.3)	60 (38.0)
Two or more	44 (67.7)	98 (62.0)
Economic situation		
Same as usual	39 (60.0)	73 (46.2)
Worse than usual	20 (30.8)	71 (44.9)
Better than usual	6 (9.2)	14 (8.9)
Communication with family		
Same as usual	28 (43.1)	58 (36.7)
Less than usual	2 (3.1)	9 (5.7)
More than usual	35 (53.8)	91 (57.6)
Communication with friends		
Same as usual	15 (23.1)	7 (4.4)
Less than usual	48 (73.8)	144 (91.1)
More than usual	2 (3.1)	7 (4.4)

All values given as n (%). Normal = GHQ-12 score < 4; Poor = GHQ-12 score ≥ 4. Abbreviation: GHQ-12 = General Health Questionnaire 12. * A total of 13 prefectures were designated as alert regions.

**Table 4:** Survey responses by mental health status.

	Normal Mental Health Status	Poor Mental Health Status	*p* Value
GHQ-12 score	2 (0–2)	7 (5–9)	<0.001
Anxiety about COVID-19	8 (6–9)	8 (7–10)	0.03
Health status	9 (8–10)	8 (5–9)	<0.001
Self-determination score	8 (6–9)	7 (5–9)	0.04
Satisfaction with daily life			
Leisure	4 (3–7)	2 (1–3)	<0.001
Study	5 (3–7)	4 (2–6)	0.001
Daily-life activities	6 (4–8)	4 (3–6)	<0.001
New activities	7 (6–9)	6 (1–8)	<0.001

All values given as the median (interquartile range). Normal = GHQ-12 score < 4; Poor = GHQ-12 score ≥ 4. All *p* values obtained by Mann–Whitney U test. Abbreviation: GHQ-12 = General Health Questionnaire 12.

**Table 5:** Risk factors associated with poor mental health status.

			*p* Value	
	Number of Respondents with Poor Mental Health/Number of Responses (%)	Adjusted OR (95%CI)	Category	Overall
Communication with friends				0.03
Same as usual	7/22 (31.8)	1 [Reference]	NA	
Less than usual	144/152 (94.7)	5.38 (1.54–18.83)	0.008	
More than usual	7/9 (77.8)	5.45 (0.69–43.32)	0.11	
Health condition		0.68 (0.55–0.83)		<0.001
Satisfaction with leisure		0.68 (0.58–0.79)		<0.001
Satisfaction with new activities		0.88 (0.78–0.99)		0.04
Adjusted for health condition, satisfaction with leisure, satisfaction with study, satisfaction with daily-life activities, satisfaction with new activities, communication with friends, communication with family, anxiety about COVID-19, self-determination score, household members, gender, age, year of university, and economic situation, as appropriate.				

Abbreviations: GHQ-12 = General Health Questionnaire 12; NA, not applicable; OR, odds ratio; CI, confidence interval. Category refers to the P value for each category vs. the reference, overall refers to the results of logistic regression analysis.

## Data Availability

The data from this study will not be published due to the need for participant anonymity and secondary analysis.
